# Protein Arginine Methyltransferase 1 and 8 Interact with FUS to Modify Its Sub-Cellular Distribution and Toxicity *In Vitro* and *In Vivo*

**DOI:** 10.1371/journal.pone.0061576

**Published:** 2013-04-19

**Authors:** Chiara Scaramuzzino, John Monaghan, Carmelo Milioto, Nicholas A. Lanson, Astha Maltare, Tanya Aggarwal, Ian Casci, Frank O. Fackelmayer, Maria Pennuto, Udai Bhan Pandey

**Affiliations:** 1 Department of Neuroscience and Brain Technologies, Istituto Italiano di Tecnologia, Genova, Italy; 2 Department of Genetics, Louisiana State University Health Sciences Center, New Orleans, Louisiana, United States of America; 3 Laboratory of Epigenetics and Chromosome Biology, Department of Biomedical Research, Institute of Molecular Biology and Biotechnology, Foundation for Research and Technology Hellas (IMBB-FORTH), University Campus, Ioannina, Greece; University of Florida, United States of America

## Abstract

Amyotrophic lateral sclerosis (ALS) is a late onset and progressive motor neuron disease. Mutations in the gene coding for fused in sarcoma/translocated in liposarcoma (FUS) are responsible for some cases of both familial and sporadic forms of ALS. The mechanism through which mutations of FUS result in motor neuron degeneration and loss is not known. FUS belongs to the family of TET proteins, which are regulated at the post-translational level by arginine methylation. Here, we investigated the impact of arginine methylation in the pathogenesis of FUS-related ALS. We found that wild type FUS (FUS-WT) specifically interacts with protein arginine methyltransferases 1 and 8 (PRMT1 and PRMT8) and undergoes asymmetric dimethylation in cultured cells. ALS-causing FUS mutants retained the ability to interact with both PRMT1 and PRMT8 and undergo asymmetric dimethylation similar to FUS-WT. Importantly, PRMT1 and PRMT8 localized to mutant FUS-positive inclusion bodies. Pharmacologic inhibition of PRMT1 and PRMT8 activity reduced both the nuclear and cytoplasmic accumulation of FUS-WT and ALS-associated FUS mutants in motor neuron-derived cells and in cells obtained from an ALS patient carrying the R518G mutation. Genetic ablation of the fly homologue of human PRMT1 (DART1) exacerbated the neurodegeneration induced by overexpression of FUS-WT and R521H FUS mutant in a Drosophila model of FUS-related ALS. These results support a role for arginine methylation in the pathogenesis of FUS-related ALS.

## Introduction

Amyotrophic lateral sclerosis (ALS) is a devastating neuromuscular disorder characterized by the progressive and rapid loss of upper and lower motor neurons in the cortex, brainstem, and spinal cord, together with skeletal muscle wasting, atrophy and paralysis [1], [2], [3]. The course of disease is fatal within 1 through 5 years from diagnosis due to failure of respiratory muscles. ALS has an average age at onset of around 60 years and incidence of disease is about 1-2/100,000. Although the vast majority of ALS cases are sporadic (sALS), about 10% of cases are familial (fALS), with a typical autosomal dominant pattern of inheritance, even though some recessive forms have also been described. To date, mutations in an increasing number of genes have been linked to ALS [4].

Mutations in genes coding for proteins involved in DNA/RNA metabolism, such as fused in sarcoma/translocated in liposarcoma (FUS/TLS, which we will refer to hereafter as FUS) [5], [6], [7], and the 43 kDa transactive response-DNA binding protein (TDP43) [8], [9], [10], [11], have emerged as a leading cause of ALS [12] and other motor neuron diseases [13]. Mutations in FUS are responsible for 5% of fALS cases and about 1% of sALS cases. FUS belongs to the TET protein family, which also includes Ewing's sarcoma (EWS) and TATA-binding protein-associated factor 15 (TAF15) [14]. Similar to the other members of the TET family, FUS is composed of an amino-terminal domain enriched in glutamine, glycine, serine, and tyrosine residues (QGSY-rich region), multiple regions rich in arginine and glycine residues (RGG), an RNA-recognition motif (RRM), and a very well conserved carboxy-terminal region, which contains a zinc finger motif and a nuclear localization signal. Most of the mutations causing ALS lie in the glycine-rich region and the carboxy-terminal domain, and several missense mutations involve substitution of one of the five arginine residues present in this region. FUS is a ubiquitous protein that predominantly localizes to the nucleus in neurons and glial cells [15]. ALS-linked FUS mutants abnormally distribute to cytoplasm, where they accumulate into stress granules [16], [17], [18], in an RNA-dependent manner [19]. In a fly model of FUS-related ALS, deletion of the nuclear export signal blocks the accumulation of mutant FUS in the cytoplasm resulting in the absence of toxicity, further supporting the notion that mislocalization of mutant FUS to cytosol is critical for toxicity [20].

TET proteins' function and biology is regulated at the post-translational level by arginine methylation [14]. Arginine methylation is accomplished by a family of proteins, namely protein arginine methyltransferases (PRMTs) [21], [22], [23], [24]. Mammalian cells express at least eight PRMTs, named PRMT1, 2, 3, 4, 5, 6, 7, and 8. PRMTs transfer a methyl group from the donor molecule S-adenosyl-L-methionine (AdoMet) to the terminal nitrogen atom of the guanidinium side chain of the arginine residues of a target protein. Arginine residues contain one internal δ-guanidino nitrogen atom and two ω-guanidino nitrogen atoms. Arginine residues can be monomethylated or dimethylated, and dimethylation can be both asymmetric (ADMA), when two methyl groups are added to the same guanidino nitrogen, or symmetric (SDMA), if one methyl group is added to each guanidino nitrogen. ADMA is catalyzed by the type I class of PRMTs, which includes PRMT1, 3, 4, 6, and 8, and SDMA is catalyzed by type II class, which includes PRMT5 and PRMT7. FUS has been shown to be predominantly asymmetrically dimethylated [25]. Recently, FUS has been shown to physically and functionally interact with and be arginine-methylated by PRMT1 [26], [27]. Importantly, arginine methylation by PRMT1 has been shown to regulate FUS subcellular localization in physiological and pathological conditions [28], [29]. PRMT1 and PRMT8 share 80% homology and have similar catalytic activity, but different from PRMT1, PRMT8 is myristoylated [30]. PRMT8 is expressed in the central nervous system (CNS) and not in peripheral tissues, and, importantly, in the CNS PRMT8 is highly and selectively expressed in brain and spinal cord, suggesting a critical role of PRMT8 in neurons [30], [31], [32]. However, whether PRMT8 interacts with FUS and plays a role in FUS-related ALS pathogenesis had not been characterized.

Here, we investigated the impact of arginine methylation and PRMTs function in the pathogenesis of FUS-related ALS in mammalian cell culture, ALS patient cells carrying a disease-causing mutation in FUS, and in a Drosophila model of FUS-related ALS. Here, we show that both FUS-WT and ALS-associated FUS mutants form a complex with PRMT1 and PRMT8 and undergo asymmetric dimethylation. PRMT1 and PRMT8 localized to FUS-positive inclusion bodies. Pharmacologic inhibition of PRMT function reduced the cytoplasmic mislocalization of FUS mutants. Moreover, genetic ablation of the PRMT1 and PRMT8 fly ortholog enhanced the neurodegeneration in a fly model of FUS-related ALS. These results provide the first evidence that PRMT1 and PRMT8 modify ALS pathogenesis *in vivo*.

## Materials and Methods

### Plasmids and reagents

Wild type and mutant FUS constructs were a generous gift from Dr. Christopher Shaw (King's College, London, UK). Adenosine dialdehyde (Adox, A7154, Sigma) and AMI-1 (Cat #539209, Calbiochem) were dissolved in DMSO.

### Cell cultures and transfections

Motor neuron-derived (MN-1) cells [33], COS1 (ATCC, CRL-1650), and human embryonic kidney 293 T (HEK293T, ATCC,CRL-1573) cells were cultured as previously described [34]. COS1 cells (1×10^6^) were transiently transfected using Lipofectamine 2000 (Invitrogen). HEK293T were transfected using Lipofectamine/Plus reagent (Invitrogen). An Epstein-Barr immortalized lymphoblastoid cell line carrying the FUS-R518G mutation and an age- and gender-matched control lymphoblastoid line were obtained from the NINDS Repository at the Coriell Institute for Medical Research (ND14136 and ND00066, Camden, New Jersey). The FUS-R518G mutant cell line was verified by sequencing the PCR product obtained using the forward primer 5′-CTAGGCTTGGAGAGGCTGG and reverse primer 5′-GGGCAAATTTAGGCCAACAC. Control and FUS-R518G lymphoblastoid cells were grown in Advanced DMEM (Invitrogen) supplemented with 10% FBS and 2 mM GlutaMax-1 (Invitrogen).

### Immunocytochemistry

Immunofluorescence in COS1 cells was performed as previously described [34], [35]. Primary antibodies were: anti-HA (1∶200, Santa Cruz, sc-805), anti-FUS (1∶1000, A300-302A, Bethyl Labs, and sc-25540, Santa Cruz); anti-enhanced green fluorescent protein (EGFP, Roche). Secondary antibodies were: Alexa Fluor 546-Goat Anti-Rabbit IgG and Alexa Fluor 488-Goat Anti-Rat IgG (Invitrogen). Lymphoblastoid cells were fixed in 3% formaldehyde in PBS for 15 minutes at room temperature. Cells were washed in PBS and permeabilized by incubation in PBS containing 0.1% Triton X-100 with 5% normal goat serum (Invitrogen) for 1 hour at room temperature. Cells were incubated with anti-FUS and goat anti-rabbit AlexaFluor 488 secondary antibodies in PBS, 0.1% Triton X-100 and 5% normal goat serum. DAPI and DRAQ5 (Biostatus Limited) were used for nuclear staining. Cells were embedded in Prolong Gold medium (Invitrogen). Images were acquired digitally with a NIKON Eclipse 80i upright microscope. Quantification of cells with nuclear and cytosolic FUS was performed as follows: Cells were classified into five groups: cells with FUS in the nucleus, more in the nucleus than in the cytosol, equally divided between nucleus and cytosol, more in the cytosol, or only in the cytosol.

### Western blotting, immunoprecipitation, and nuclear/cytosolic fractionation

For Western blotting analysis, cells were washed with ice-cold PBS and scraped in 100 µl lysis buffer (150 mM NaCl, 2% sodium dodecyl sulfate, 10 mM Hepes pH 7.4, 2 mM EDTA) plus protease inhibitor cocktail (Roche Diagnostics). Total lysates were sonicated and centrifuged at 13000 rpm for 10 min at 4°C. Cells lysates were denatured at 95°C in 5× sample buffer (1× final concentration is 60 mM Tris, pH 6.8, 2% SDS, 25% glycerol, 0.1% bromophenol blue, 20% b-mercaptoethanol) and processed for 7.5–10% sodium dodecyl sulfate–polyacrylamide gel electrophoresis (SDS–PAGE), and electro-transferred onto nitrocellulose membranes (Millipore). Immunoblotting was done in 5% non-fat dry milk dissolved in Tris-buffered saline using the following antibodies: FUS (1∶500, sc-25-540, Santa Cruz), α-Tubulin (1∶10,000, Sigma #T5168), EGFP (1∶1000, A10262, Invitrogen); asymmetric dimethyl-arginine ASYM24 (1∶500, 07-414, Millipore), HA (1∶1000, 11095200, Roche Diagnostics), and c-JUN (1∶1000, ab1964, Abcam). Immunoreactivity was detected using peroxidase-conjugated AffiniPure Goat Anti-Rabbit or Anti-Mouse IgG (Jackson ImmunoResearch), and visualized using LIGHTNING chemiluminescence reagent (Perkin-Elmer) following the manufacturer's instructions.

All immunoprecipitation (IP) procedures were carried out at 4°C. HEK293T cells were washed with ice-cold PBS, scraped in 500 µl IP buffer (50 mM HEPES, 250 mM NaCl, 5 mM EDTA, 0.1% Nonidet P-40) plus protease inhibitor cocktail (Roche Diagnostics) and sonicated. Cleared lysates were immunoprecipitated using anti-HA or anti-EGFP antibodies for 3 hours at 4°C. Immunoprecipitated proteins were then washed three times in IP buffer, resuspended in sample buffer, boiled, and subjected to 10% SDS–PAGE. Immunoblotting was done as described above. We used protein A/G plus Agarose from Santa Cruz for IP with anti-GFP, protein G Agarose from Thermo Scientific for IP with anti GFP, anti FLAG M2 affinity gel for IP with anti FLAG.

All nuclear-cytosolic fractionation procedures were carried out at 4°C according to the manufacturer's instructions (NE-PER 78833, Thermo Scientific). Samples were analyzed by SDS-PAGE as described above.

### Drosophila culture, light microscopy, quantification and qPCR

The FUS transgenic flies and GMR-gal4 driver were described previously [20]. DART1 RNAi lines (ID# 40388, 110391) were obtained from the Vienna Drosophila Research Center. Eye phenotypes of 1-day-old flies were analyzed with a Leica M205C stereomicroscope and photographed with a Leica DFC420 digital camera. For each genotype and condition, 100 to 1000 flies were evaluated.

We determined the endogenous knockdown levels of DART1 in the fly heads using qPCR methods as described previously [36]. Briefly, we determined the expression levels of DART1 and the housekeeping gene GAPDH1 using reverse transcription of mRNA purified from fly heads and QPCR with Taqman assays (Dm 02138836_g1 for DART1 and Dm 01843827_s1 for GAPDH1, Applied Biosystems). DART1 depletion in flies expressing DART1 siRNA under control of the GMR GAL4 driver was assessed by normalizing DART1 values against GAPDH1 values and comparison against control flies.

### Statistical analysis

All the experiments were replicated a minimum of three times. A one-way ANOVA and two-sample *t*-tests were used for post-hoc comparisons. A paired T-test was used to test for statistical difference in eye degeneration between fly genotypes.

## Results

### FUS-WT and ALS-linked FUS mutants selectively interact with PRMT1 and PRMT8

**Figure 1:**
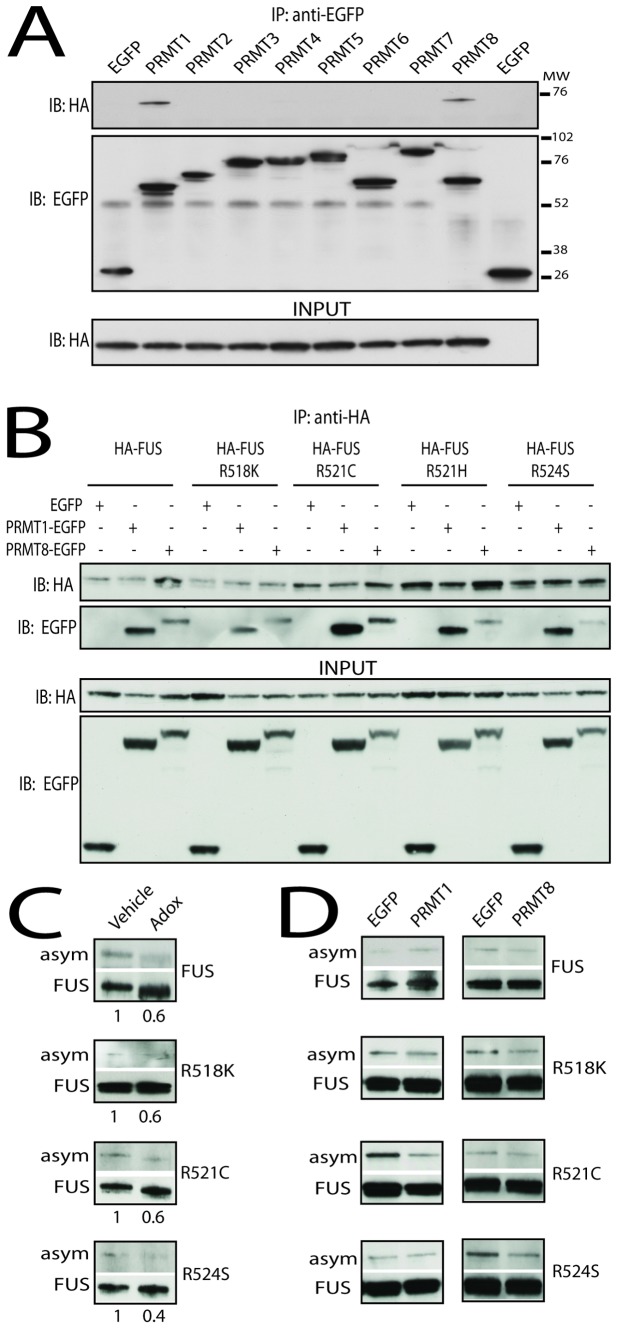
FUS-WT and ALS-linked FUS mutants selectively interact with PRMT1 and PRMT8 and undergo arginine dimethylation. A) HEK293T cells expressing HA-tagged FUS-WT and the indicated EGFP-tagged PRMTs were processed for immunoprecipitation (IP) analysis using an anti-EGFP antibody, followed by immunoblotting (IB) with anti-HA and anti-EGFP. Input of FUS is shown in the bottom panel. B) HEK293T cells expressing FUS-WT and the indicated FUS mutants together with either soluble EGFP or EGFP-tagged PRMT1 or PRMT8 were processed for IP using an anti-HA antibody and anti-EGFP IB analysis. Input is shown on bottom panel. C) HEK293T cells were transfected with either HA-tagged FUS-WT or the indicated FUS mutants and incubated with Adox for 20 hours. FUS was then immunoprecipitated with anti-HA antibody and asymmetric methylation (asym) was analyzed with a specific antibody. D) HEK293T cells were transfected with HA-tagged FUS-WT or the indicated FUS mutants together with either soluble EGFP, PRMT1-EGFP, or PRMT8-EGFP and processed for IP assay as described in (C).

Mammalian cells express at least eight PRMTs, named PRMT1-8 [21], [22]. To determine whether FUS-WT preferentially interacts with any of these PRMTs, we transiently co-transfected HEK293T cells with a vector expressing FUS-WT fused to the HA tag on the amino-terminal portion together with a vector expressing either soluble EGFP or PRMTs 1–8 fused to EGFP (**Figure 1A**). FUS and PRMT interaction was analyzed by immunoprecipitation assay using anti-EGFP antibody. We found that FUS-WT selectively and specifically interacts with PRMT1 and PRMT8. Similar results were obtained by immunoprecipitation of FUS using the anti-HA antibody and staining with the EGFP antibody (**Figure 1B** and data not shown). Moreover, the same pattern of interactions was observed with a FUS version in which the Flag tag was fused to the carboxy-terminal portion of FUS, indicating that fusion of a tag to either the amino-terminal portion or the carboxy-terminal portion of FUS does not affect its ability to interact with these PRMTs (data not shown).

We hypothesized that specific fALS-associated arginine point mutations in the carboxy-terminal portion of FUS may alter the interaction with PRMT1 and PRMT8. We tested this hypothesis using ALS-associated FUS mutants, in which either arginine 518 was mutated to lysine (R518K), arginine 521 to cysteine and histidine (R521C and R521H), or arginine 524 to serine (R524S). HA-tagged FUS-WT and the aforementioned FUS mutants were expressed in cultured cells together with either EGFP, PRMT1-EGFP, or PRMT8-EGFP (**Figure 1B**). The cells were processed for immunoprecipitation assay followed by immunoblotting analysis with anti-HA and anti-EGFP antibodies. We found that the ALS-associated FUS mutants tested here retain the ability to interact with both PRMT1 and PRMT8 in cultured cells.

PRMT1 and PRMT8 are type I arginine methyltransferases that catalyze the production of asymmetrically dimethylated arginine residues [22], [37]. In order to determine whether FUS-WT and ALS-linked FUS mutants undergo asymmetric dimethylation at arginine residues, we expressed FUS-WT and the FUS mutants in HEK293T cells, isolated FUS by immunoprecipitation and detected asymmetrically dimethylated arginine using an anti-asymmetric dimethylated arginine antibody (**Figure 1C**). The anti-asymmetric dimethylation antibody detected FUS-WT as well as the FUS mutants, indicating that these ALS-linked FUS mutants undergo asymmetric dimethylation similar to FUS-WT in cultured cells. Treatment of the cells with the methyltransferase inhibitor Adox resulted in a decrease in the asymmetric dimethylation of FUS-WT and the FUS mutants. This is consistent with previous reports that show that FUS-WT and ALS-linked FUS mutants are methylated at arginine residues, and ALS-related mutations do not alter global FUS arginine methylation [26], [29]. To address whether overexpression of PRMT1 and PRMT8 affects FUS arginine methylation, we overexpressed either PRMT1 or PRMT8 together with FUS-WT and the FUS mutants (**Figure 1D**). However, we did not observe any change in the arginine dimethylation status of FUS by overexpressing either PRMT1 or PRMT8, suggesting that endogenous PRMTs are sufficient to fully methylate FUS. All together, these findings indicate that ALS-related FUS mutants form a complex with PRMT1 and PRMT8 and undergo asymmetric dimethylation similar to FUS-WT.

### PRMT1 and PRMT8 accumulate in mutant FUS-positive inclusion bodies

**Figure 2:**
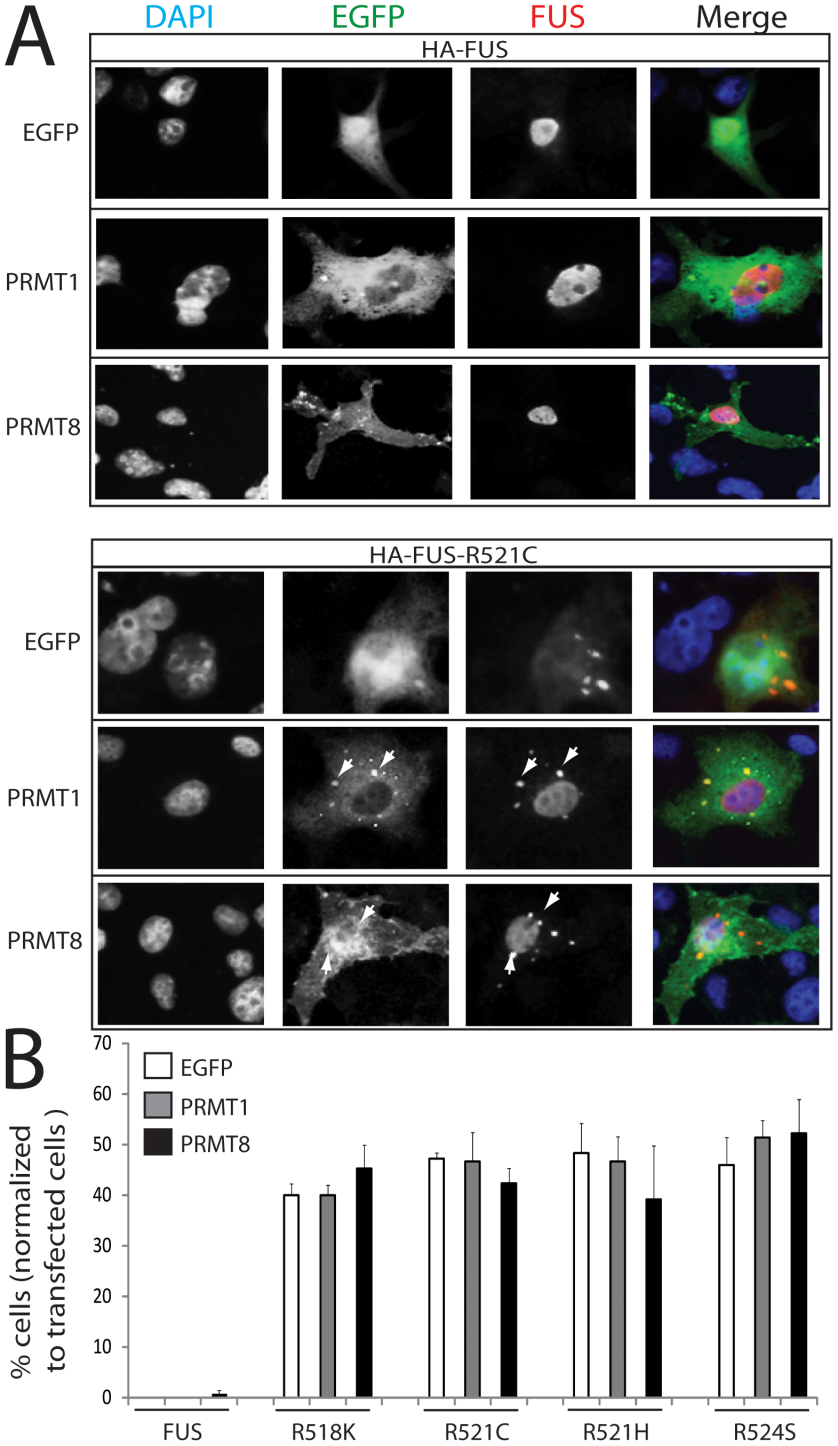
PRMT1 and PRMT8 localize to FUS-positive inclusion bodies. A) COS 1 cells were transfected with HA-tagged FUS-WT or FUS-R521C together with either EGFP, PRMT1-EGFP, or PRMT8-EGFP, and processed for immunofluorescence analysis. FUS was detected with the anti-HA antibody, and nucleus with DAPI. PRMT1 and PRMT8 localize to mutant FUS-positive inclusion bodies (arrows). B) Quantification of cells with nuclear inclusions normalized to total number of transfected cells (n = 100/sample). Graph, mean ± s.e.m.

Mutant FUS has previously been shown to accumulate in perinuclear inclusion bodies in cultured cells [17]. To assess whether PRMT1 or PRMT8 localize to FUS-positive inclusion bodies, we transfected COS1 cells with a vector expressing FUS-WT or FUS -R518K, -R521C, -R521H, or -R524S mutants tagged to HA together with either EGFP, PRMT1-EGFP, or PRMT8-EGFP, and we analyzed the subcellular distribution of FUS and the PRMTs by immunofluorescence (**Figure 2A** and **Figure S1**). As previously described [17], FUS-WT predominantly localized to the nucleus. No inclusion bodies were observed in the cells overexpressing FUS-WT. All the ALS-linked FUS mutants analyzed here localized to the nucleus, and in addition they assembled into perinuclear inclusion bodies, which resemble stress granules. PRMT1 is a soluble protein that mainly localizes to cytoplasm, while PRMT8 localizes to the membrane fraction due to myristoylation [30]. We found that FUS-WT and the FUS mutants co-localize with PRMT1 and PRMT8. Importantly, both PRMT1 and PRMT8 accumulated in inclusion bodies in the cells expressing the FUS mutants. To determine whether overexpression of PRMT1 and PRMT8 affects the deposition of the FUS mutants into inclusion bodies, we counted the number of transfected cells forming inclusion bodies. Overexpression of neither PRMT1 nor PRMT8 altered inclusion body formation in this cell type (**Figure 2B**). These results indicate that PRMT1 and PRMT8 are sequestered into mutant FUS-positive inclusions in cultured cells.

### Arginine methylation affects the sub-cellular localization of FUS-WT and ALS-linked FUS mutants in cultured cells

**Figure 3:**
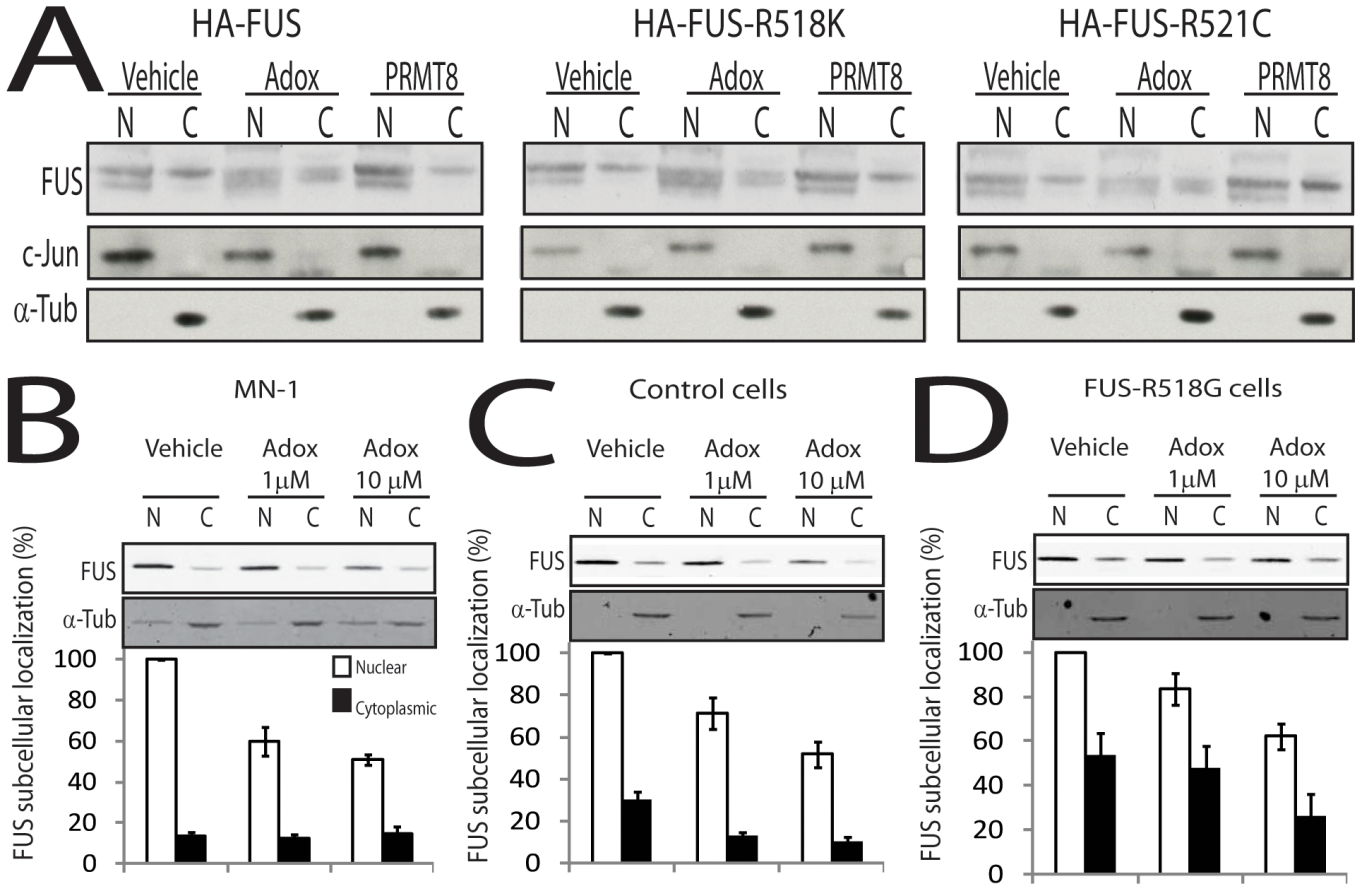
Arginine methylation affects the sub-cellular localization of mutant FUS in cultured cells. A) HEK293T cells were transfected with FUS-WT or the indicated FUS mutants, together with EGFP or PRMT8-EGFP, and treated with vehicle or Adox (10 µM) for 24 hours. The cells were then subjected to nuclear/cytoplasmic fractionation, and the nuclear (N) and cytosolic (C) fractions were analyzed by Western blotting. c-JUN and alpha-tubulin were used as loading controls of nuclear and cytosolic fractions, respectively. B) MN-1 Motor neuron cells were treated with 1 and 10 µM Adox for 24 hours. Proteins from the nuclear and cytoplasmic fractions were analyzed by western blotting with anti-FUS antibody. Alpha-tubulin is shown as loading control. Quantification is shown in bottom panel. Graph, mean +/− s.e.m. C) Nuclear and cytoplasmic fractionation of lymphoblastoid cells derived from normal control analyzed as described in (B). D) Nuclear and cytoplasmic fractionation of lymphoblastoid cells derived from an ALS patient in which FUS carried the R518G mutation.

PRMTs are known to regulate the nuclear transport of RNA binding proteins [38], [39]. Because the subcellular localization of FUS is critical in ALS pathogenesis [17], [20] we reasoned that the interaction of FUS with PRMTs is important for the subcellular localization of FUS. Using nuclear/cytoplasmic fractionation, we analyzed the sub-cellular distribution of FUS-WT and the R518K and R521C FUS mutants in HEK293T cells treated with Adox and in cells overexpressing PRMT8 (**Figure 3A**). Treatment of the cells with Adox resulted in a slight reduction in the accumulation of endogenous FUS not only in the nucleus, but also in the cytosol. Notably, we observed a reduction in the total levels of FUS in the Adox-treated cells, indicating that PRMT inhibition reduces the accumulation of the protein. Overexpression of PRMT8 had the opposite effect, as it resulted in an increase in the accumulation of FUS in both the nucleus and cytosol.

Next, we sought to determine whether arginine methylation regulates the distribution of endogenous FUS in motor neuron-derived MN-1 cells (**Figure 3B**). Treatment of the cells with Adox resulted in a significant reduction in the cytoplasmic levels of FUS. Similar to what is observed in HEK293T cells, Adox treatment also reduced the accumulation of endogenous FUS in the MN-1 cells. These results indicate that inhibition of arginine methylation results in a reduced accumulation of FUS-WT and ALS-linked FUS mutants in cultured cells.

### Inhibition of PRMT function decreases the cytosolic accumulation of R518G FUS mutant in ALS patient-derived cells

**Figure 4:**
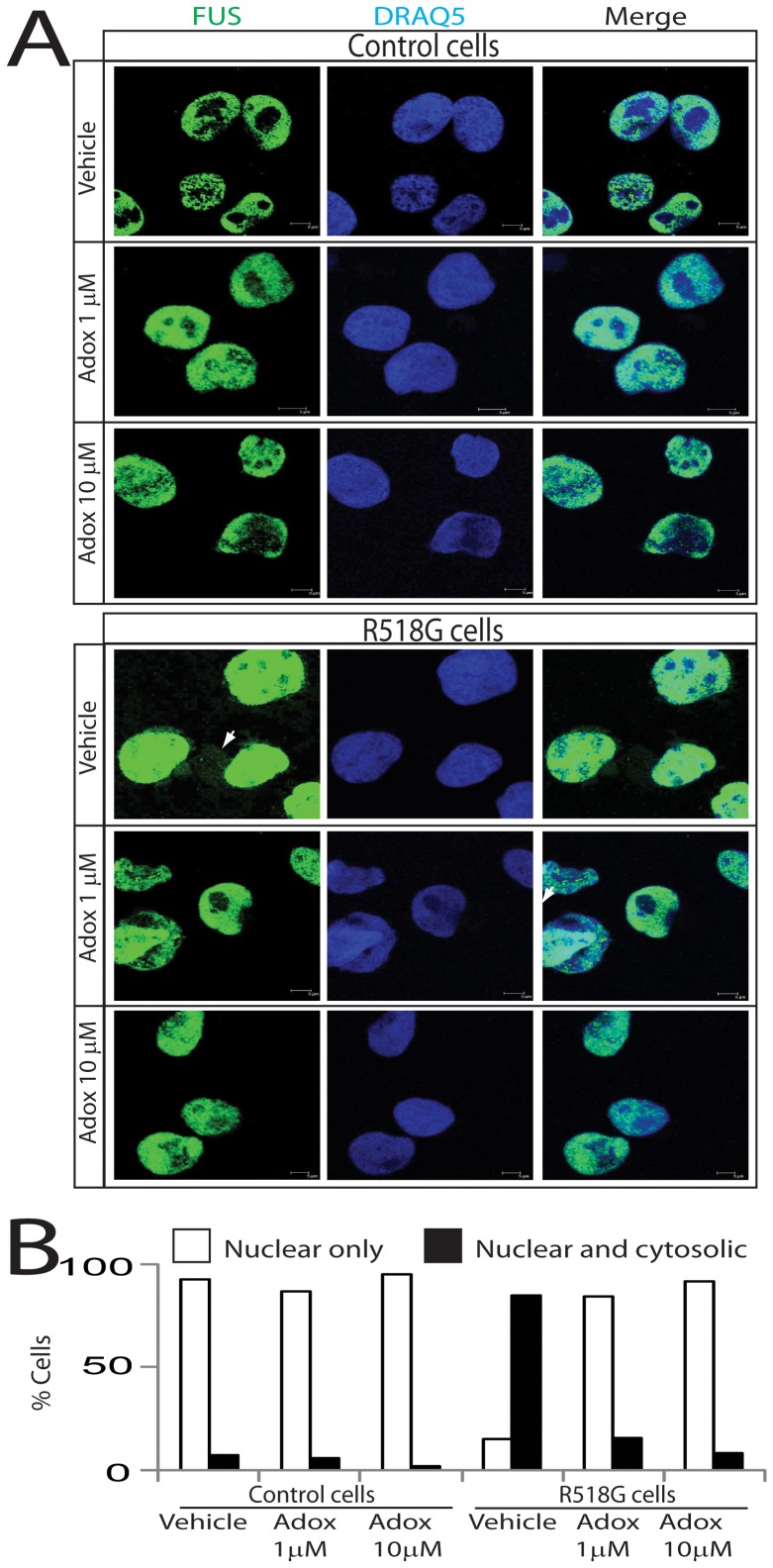
Treatment with Adox reduces cytosolic accumulation of mutant FUS in patient cells carrying the mutation R518G. A) Cells from an ALS patient with the FUS R518G mutation and a control individual were treated with 1 or 10 µm Adox for 24 hours and stained with both anti-FUS (green) and DRAQ5 (nuclei, blue). B) Control and R518G mutant cells treated with vehicle and Adox were scored for the presence of FUS only in the nucleus or in both the nucleus and cytoplasm (n = 100 cells were counted for each sample).

**Figure 5:**
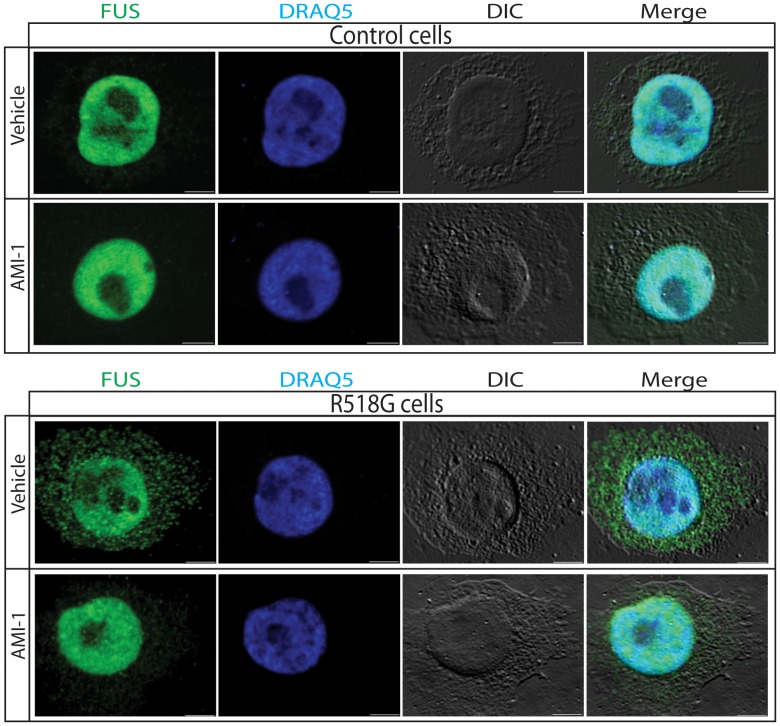
Treatment with the PRMT-1 specific inhibitor AMI-1 reduces mutant FUS accumulation in the cytosol of FUS-R518G patient-derived mutant cells. Patient-derived lymphoblastoid cells and the control line were treated with vehicle or 150 µm AMI-1 for 24 hours and stained with both anti-FUS and DRAQ5 (nuclear stain).

In order to determine whether the reduced nuclear accumulation of normal and mutant FUS observed upon inhibition of arginine methylation is relevant in ALS pathogenesis, we used a human lymphoblastoid cell lines carrying the R518G mutation obtained from an ALS patient and cells from an age-matched control (**Figure 3C and D**). We observed almost equal FUS protein expression in the cells expressing FUS R518K and control cells (**Figure S2**). We found that Adox treatment decreases the accumulation of FUS in the nucleus of control and mutant cells. Notably, the R518G lymphoblasts had more than twice as much FUS in the cytoplasm as the normal lymphoblasts. We also analyzed the subcellular localization of FUS-WT and FUS-R518G in response to Adox treatment by immunofluorescence in control and patient-derived cells (**Figure 4A**). As expected, endogenous FUS-WT mostly localized to nucleus, whereas FUS-R518G was distributed in both the cytoplasm and nucleus. Importantly, Adox treatment reduced the accumulation of endogenous FUS-R518G in the cytosol. To quantify the effect of Adox, we counted the cells with FUS only in the nucleus or in both nucleus and cytosol (**Figure 4B**). In normal cells, over 95% of FUS-WT localized in the nucleus independently of Adox treatment. In the patient-derived cells, only 15% of the cells contained FUS-R518G only in the nucleus. Treatment of the mutant cells with Adox restored the nuclear localization of FUS-R518G in over 95% of the cells. To determine whether the subcellular localization of FUS-R518G is regulated by PRMT1, we treated the cells derived from normal controls and ALS patients with the PRMT-1 specific inhibitor AMI-1 (**Figure 5**). Treatment of the mutant cells with AMI-1 decreased the cytoplasmic localization of FUS-R518G, further implying PRMT1 function in ALS pathogenesis.

### Genetic ablation of PRMT1 enhances mutant FUS-induced degeneration in flies

**Figure 6:**
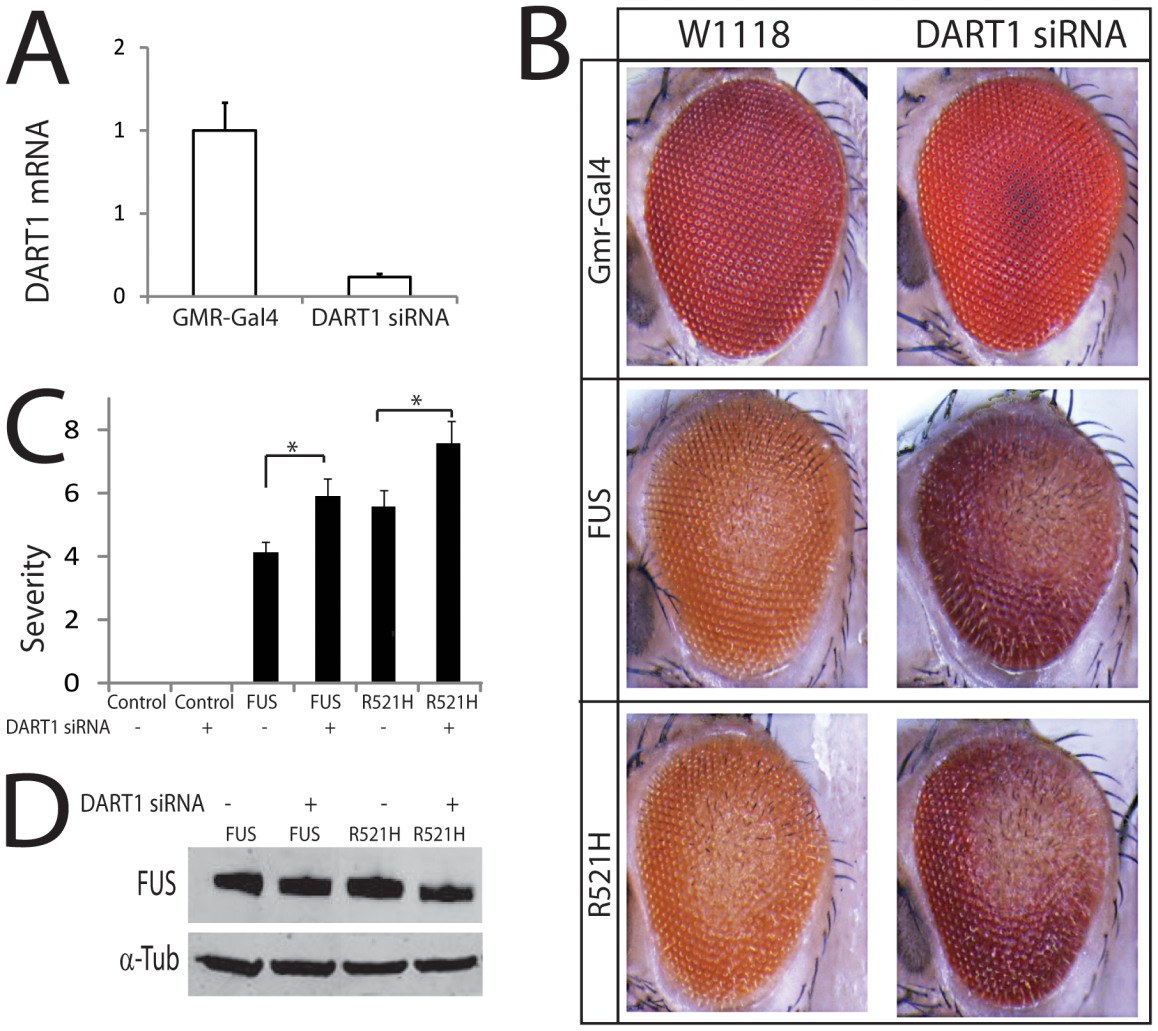
PRMT1 knock down enhances degeneration in a fly model of FUS-related ALS. A) Real-time PCR analysis of DART1 mRNA transcript levels in Drosophila revealed 80% knockdown of DART1 mRNA in RNAi transgenic lines as compared to control flies. B) Genetic deletion of DART1 in the fly eyes expressing either FUS-WT or FUS-R521H mutant enhanced the external eye degeneration caused by FUS C) Quantification of eye phenotype (see “Material and Methods” section). D) Western blotting analysis of FUS levels in the eye of DART1 knock down and control lines.

The observation that PRMT1 and PRMT8 interact with FUS-WT and ALS-linked FUS mutants and localize to mutant FUS-positive stress granules led us to hypothesize that the PRMT-FUS interaction may play a role in ALS pathogenesis. To investigate the biological significance of the interaction of FUS with PRMT1 and PRMT8, we used a Drosophila model of FUS-related ALS that we recently developed and that recapitulates several key features of human ALS, such as mutation-dependent toxicity, mislocalization of mutant FUS into the cytoplasm, and behavioral defects [20]. As we previously described, ectopic expression of FUS-WT resulted in mild eye degenerative phenotype, whereas expression of FUS-R521H caused severe external eye degeneration. We assessed the effect of PRMT1 on FUS-induced degeneration, using a UAS-RNAi line to knock down endogenous expression of DART1 - the single ortholog of both PRMT1 and PRMT8 in the fly - in the Drosophila eye. First of all, we verified that the line expressing DART1 RNAi had reduced expression of DART1 mRNA transcript levels by real-time PCR analysis (**Figure 6A**). Depletion of endogenous DART1 alone did not cause any obvious external eye phenotype in Drosophila (**Figure 6B**) but genetic ablation of DART1 enhanced the neurodegenerative phenotype induced by FUS-WT and FUS-R521H, as evident from the increase in the area showing external eye degeneration in the fly eyes expressing either FUS-WT or FUS mutant together with DART1 RNAi. To quantify the effect of DART1 ablation on disease severity, we scored disease severity as previously described [20], [35], [36] (**Figure 6C**). The effect of DART1 deletion was not associated with any change in FUS expression (**Figure 6D**). These data provide evidence that *in vivo* loss of PRMT1 and PRMT8 function enhances mutant FUS toxicity, indicating a primary role for PRMT1 and PRMT8 in FUS-related ALS pathogenesis.

## Discussion

Here, we show that FUS-WT and fALS-related FUS mutants selectively interact with PRMT1 and PRMT8. We provide evidence that PRMT1 and PRMT8 localize to cytosolic inclusions formed by mutant FUS. We show that PRMT function regulates the subcellular distribution of FUS-WT and FUS mutants in motor neuron-derived cells and in lymphoblastoid cells derived from an fALS patient carrying the R518G mutation. Finally, we show that in a fly model of FUS-related ALS, loss of PRMT1 and PRMT8 enhances the degenerative phenotype, highlighting a genetic and functional interaction between FUS and PRMT1 and PRMT8 *in vivo*. Our results provide evidence that PRMT1 and PRMT8 functions play a critical role in ALS pathogenesis.

Intracellular and extracellular aggregation and deposition of misfolded protein are hallmarks of many human neurodegenerative diseases, including Alzheimer's disease, Parkinson's disease, frontotemporal lobar degeneration, polyglutamine diseases, and ALS [40], [41]. Although these disorders have distinct individual clinical and neuropathological features, they share common aspects, including late onset, and sporadic as well as familial patterns of inheritance. One important aspect of these diseases are lesions in the central nervous system that result from the accumulation of misfolded proteins in forms of ubiquitinated micro-aggregates/oligomers and inclusions, species to which neurons seem to be particularly sensitive. Micro-aggregates are detectable by biochemistry, and inclusions are visualized by immunofluorescence techniques. Inclusion formation in polyglutamine diseases have been shown to be protective in several models of polyglutamine diseases, such as Huntington's disease [42] and spinal and bulbar muscular atrophy [35]. On the other hand, accumulation of misfolded protein into micro-aggregates or oligomers has been largely correlated to cytotoxicity. FUS-positive inclusions have been detected in non-SOD1 ALS patient specimens, frontotemporal lobar degeneration, and neuronal intermediate filament inclusion disease [43], [44], [45]. Accumulation of FUS and TDP43 into inclusions is a common feature of ALS and other diseases caused by protein misfolding, suggesting that FUS and TDP43 pathology have a broad impact. In cultured cells, mutant FUS accumulates in inclusion bodies, which have been identified as stress granules [17]. The role of stress granules in disease pathogenesis is not known. Ubiquitin-positive FUS aggregates have been found in fALS [46] and in specific cases of frontotemporal lobar degeneration [47]. Because overexpression of PRMT1 and PRMT8 did not affect the deposition of mutant FUS into inclusion bodies, arginine methylation by these PRMTs does not seem to affect this aspect of pathogenesis.

Arginine methylation has a critical impact on the subcellular localization and function of the TET proteins. Arginine methylation of EWS by PRMT1 increases the accumulation of the protein in the cytosol and alters protein function [48], while arginine methylation of TAF15 and FUS by PRMT1 has the opposite effect on protein function [26], [49]. Interestingly, we found that ALS-related FUS mutants did not alter either the ability of the disease proteins to interact with PRMT1 or PRMT8 or the overall methylation status of the proteins, indicating that substitutions of arginine residues in the carboxy-terminal portion of FUS does not compromise arginine methylation. This is consistent with previous findings showing that ALS-related FUS mutants undergo asymmetric dimethylation similar to FUS-WT [29].

FUS and TDP43 are RNA binding proteins that mainly localize to the nucleus in neuronal and non-neuronal cells, and they shuttle in association with RNA from the cytosol to the nucleus. FUS and TDP43 are involved in RNA metabolism, processing, and splicing, and are associated with several RNA binding proteins. FUS and the other TET proteins have pleiotropic functions in cells. TET proteins bind both DNA and RNA and regulate cellular homeostasis and gene expression at several levels [14]. TET proteins regulate DNA repair and are involved in genomic stability. Knock down of FUS leads to genomic instability in mice [50], [51]. TET proteins are associated with the RNA polymerase II transcriptional machinery and the splicing machinery [52]. ALS-linked point mutations in the carboxy-terminal portion of FUS alters the trafficking of the protein and leads to accumulation of mutant FUS in stress granules [16], [17], [53]. Some FUS mutants alter splicing regulation [18]. The mechanism through which fALS-related FUS mutants results in motor neuron degeneration is not known. It is clear that these FUS mutants mislocalize to the cytosol and accumulate into perinuclear stress granules. The subcellular mislocalization may result in a loss of protein function in the nucleus as well as a toxic gain of function in the cytosol. Localization of the PRMTs in FUS-positive inclusion bodies may result in sequestration and loss of PRMT function.

PRMT activity results in a change in subcellular distribution of FUS-WT and ALS-linked FUS mutants. We show here that inhibition of PRMT activity using the general methylation inhibitor Adox results in decreased nuclear accumulation of FUS-WT and FUS mutants. Tradewell and colleagues have recently reported that PRMT1 modulates the subcellular localization of FUS [28], and that PRMT1 knock down in motor neuron primary cultures increases the accumulation of mutant FUS to the cytosol as well as the deposition of the protein into stress granules. We found that knock down of PRMT1 in a fly model of ALS enhances neurodegeneration. Together, these observations support a critical role for PRMT1 in FUS-related ALS pathogenesis.

## Supporting Information

Figure S1 **PRMT1 and PRMT8 localize to FUS-positive inclusion bodies.** COS1 cells were transfected with FUS-R518K or FUS-R524S together with either EGFP, PRMT1-EGFP, or PRMT8-EGFP. The cells were then processed for immunofluorescence. PRMT1 and PRMT8 localize to mutant FUS-positive inclusion bodies (arrows).
(TIF)

Figure S2 **FUS protein expression level in a human ALS patient cell carrying FUS R518G mutation and age/sex matched control line.**
(TIF)
