# Supplementary figures and images for: Protein Arginine Methyltransferase 1 and 8 Interact with FUS to Modify Its Sub-Cellular Distribution and Toxicity In Vitro and In Vivo
Source: PLoS One. 2013 Apr 19;8(4):e61576. doi: 10.1371/journal.pone.0061576 (PMC3631215; doi:10.1371/journal.pone.0061576)

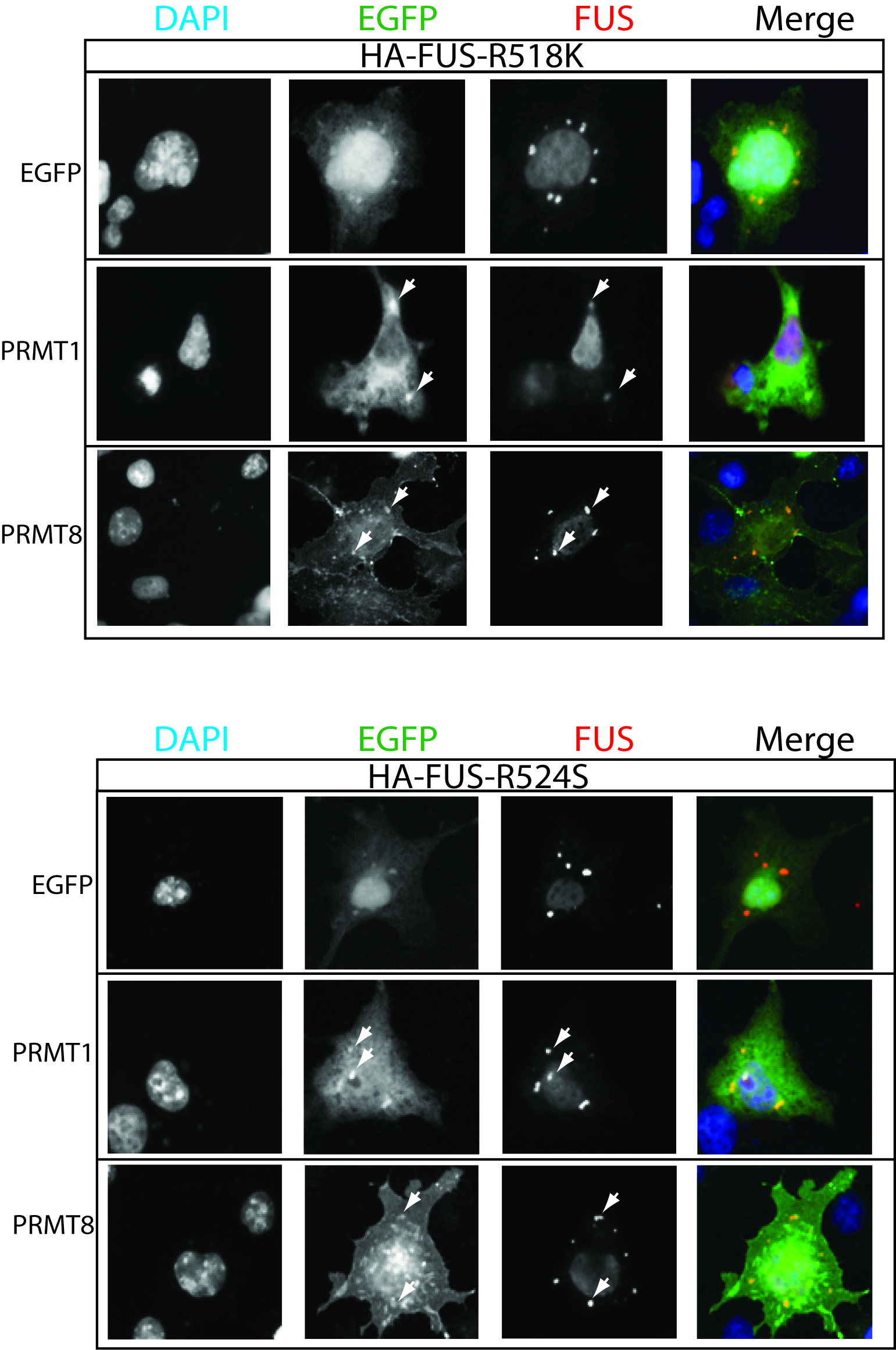

Supplement: Figure S1 — PRMT1 and PRMT8 localize to FUS-positive inclusion bodies. COS1 cells were transfected with FUS-R518K or FUS-R524S together with either EGFP, PRMT1-EGFP, or PRMT8-EGFP. The cells were then processed for immunofluorescence. PRMT1 and PRMT8 localize to mutant FUS-positive inclusion bodies (arrows). (TIF) [file pone.0061576.s001.tif]

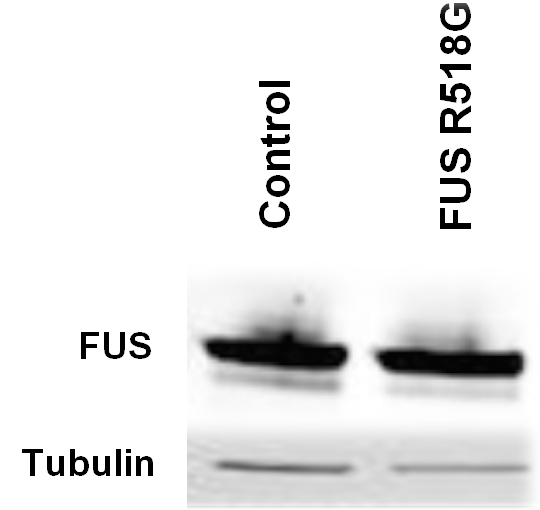

Supplement: Figure S2 — FUS protein expression level in a human ALS patient cell carrying FUS R518G mutation and age/sex matched control line. (TIF) [file pone.0061576.s002.tif]
